# Can programmatic inputs improve adolescent mothers’ access to maternal care in rural Bangladesh? Nine years of evidence from a cohort study

**DOI:** 10.1186/s41043-022-00289-8

**Published:** 2022-03-28

**Authors:** Aminur Rahman, Tahmina Begum, Anne Austin, Md. Hasan, Nurul Alam, Iqbal Anwar, Surasak Taneepanichskul

**Affiliations:** 1 Health System and Population Studies Division, International Centre for Diarrheal Disease Research (Icddr,b), 68 Shaheed Tajuddin Ahmed Sarani, Mohakhali, Dhaka 1212 Bangladesh; 2 JSI Research & Training Institute, Inc., Boston, MA USA; 3 Department of Public Health and Informatics, Bangabandhu Sheikh Mujib Medical University, Dhaka, Bangladesh; 4 College of Public Health Sciences, Chulalongkorn University, Bangkok, Thailand

**Keywords:** Adolescent pregnancy, Antenatal care, Facility delivery, Maternal health, Bangladesh

## Abstract

**Background:**

Adolescent mothers (Girls aged 15–19) constitute 8% of annual global births, but account for 10% of annual maternal deaths. WHO recommended 4–8 Antenatal Care (ANC) visits, in addition to quality care and facility-based deliveries, are well-documented interventions to reduce maternal and child morbidity and mortality. Determinants of maternal and child health care in Bangladesh have received considerable attention, but less attention has been focused on adolescent mothers. This study explores the factors associated with 4 or more (4 +) ANC visits and facility-based delivery among adolescent mothers in one rural area of Bangladesh.

**Methods:**

This study uses Health and Demographic Surveillance System (HDSS) data. We conducted a comparative study on trends in 4 + ANC visits and facility-based deliveries among adolescent mothers (10–19 years) residing in an intervention area (icddr,b service area, ISA) against a comparison area (government service areas, GSA) of HDSS between 2007 and 2015. Totally, 4,996 adolescent mothers were included in the final analysis. Binary logistic regression was used to document the statistical difference on outcome indicators in the two study areas.

**Results:**

Trends in 4 + ANC visits and facility-based deliveries were higher in the ISA relative to the GSA. The adjusted odds of an adolescent mother accessing 4 + ANC visits in the GSA, relative to ISA, were 0.57 (95% CI 0.49–0.66, *p* value < 0.05); the adjusted odds of an adolescent mother accessing facility-based delivery in the ISA, relative to GSA, were 6.63 (95% CI: 5.85–7.52, *p* value < 0.05). Increasing numbers of ANC visits were associated with increases in facility-based births in both the ISA and GSA.

**Conclusion:**

This study documented that both 4 + ANC visits and facility delivery rates among adolescent mothers are much higher in the ISA than GSA. Increasing 4 + ANC visits and facility deliveries over the years, particularly in the ISA, coincide with programmatic efforts to improve the quality and availability of maternal and newborn health services. Learning from existing interventions in ISA and applying them to other areas will strengthen Bangladesh’s efforts to improve maternal and newborn health outcomes and achieve the Sustainable Development Goal 3 (SDG 3).

## Introduction

### Global child birth and adolescent

Adolescent childbearing remains a global concern due to increased health risks and related socioeconomic consequences. Adolescent pregnancy is associated with pregnancy complications including anemia, caesarean delivery, adverse pregnancy outcomes such as premature birth, low birth weight, perinatal mortality and an increased incidence of infant mortality and morbidity [1, 2]. Girls aged 15–19 years account for 10% of global annual maternal deaths, which is disproportionate, as births to adolescent mothers constitute around 8% of all births. The burden is more intense in lower-to-middle-income countries (LMIC) where about 70, 000 adolescents die due to pregnancy and childbirth-related conditions each year [3].

### Health service accessibility

Poor access to maternity care has been highlighted as one reason for pregnancy-related mortality and morbidity among adolescent mothers, particularly in LMIC [4]. WHO recommends 8 Antenatal Care (ANC) visits for all pregnant women [5]. High-quality ANC care, combined with facility-based deliveries, are well-documented interventions that reduce maternal and child morbidity and mortality [6]. Attending periodic ANC initiates opportunities to identify and treat pregnancy risk factors and prepare mothers to attend health facilities for safe delivery [7]. Access to any maternal health services, regardless of maternal age, is low in developing countries. Pregnant women, particularly adolescent mothers, residing in rural regions are less likely to access maternal care [8]. In addition to the availability and accessibility of maternal health services, poor access to care among adolescent mothers has been attributed to their lack of social support, low female autonomy, financial barriers, and a lack of decision-making power [9–11].

### Bangladesh and adolescent pregnancy

Over the past three decades, unlike other low-income countries, Bangladesh has made significant progress on improving maternal health care indicators; the maternal mortality ratio (MMR) has declined from 320 to 176 per thousand live births, and the total fertility rate (TFR) also declined from 3.0 to 2.3[12]. However, adolescent childbearing remains a persistent problem for the nation with the highest fertility rate in South East Asia. In Bangladesh, one in ten girls has a child before the age of 15 and one in three adolescents becomes a mother or pregnant by the age of 19 [13]. Child marriage has traditionally been the leading cause of pregnancies among adolescent girls in Bangladesh [14]. The most recent (2014) Bangladesh Demographic Health Survey (BDHS) data show that among adolescent mothers, about 20% did not receive any antenatal care while 58% of deliveries took place at home without assistance from skilled attendants [15].

### Rationale

In line with the global agenda on Sustainable Development Goal (SDG) by 2030, Bangladesh has a renewed focus on adolescents as it as a crucial phase of life in the continuum of care approach [16]. To accomplish the SDG 3.1 and 3.7, "decrease Maternal Mortality Proportion to 70 for every 100,000 live births," adolescent mother should get unique care regarding accomplish the SDG [17]. This study explores the factors that were significantly associated with 4 + ANC visits and facility delivery among adolescents’ mothers in Matlab, Bangladesh. Additionally, the effects of a maternal health program on increasing access to health care services among adolescent mothers are measured and compared with an area which is solely under the standard government initiatives [18]. The particular advantage of this study is that it uses longitudinal data that provides accurate estimations of adolescent age in both the intervention and government areas as age is calculated from the date of birth of each participant. These study findings have the potential to help policymakers, programmers and researchers make informed decisions on how investments in improved access to maternal health services for adolescent mothers could contribute to overall improvement in maternal health care in Bangladesh.

## Methodology

### Study design

A retrospective longitudinal study using data from the Health and Demographic Surveillance System (HDSS) is run by the International Centre for Diarrhoeal Disease Centre (icddr,b), to analyze access to ANC care among adolescent mothers in both the icddr,b and government areas. The overall aim was to explore the determinants of and compare trends in facility delivery and 4 + ANC visits among adolescent mothers in the icddr,b intervention and government intervention areas.

### Study population

Women, in the HDSS database, who gave birth below the age of 20 years between 2007 and 2015 were the study population. In total 5,774 adolescent mothers, who gave birth between the ages of 10 and 19 years were identified. Availability of complete data on ANC care and delivery locations as well as pregnancy lasting >  = 28 weeks gestation were the major inclusion criteria. This resulted in a final sample of 4996 (87% of identified women who gave birth between the ages of 10 and 19) had complete data; 2892 from icddr,b Service Area (ISA) and 2104 from Government Service Area (GSA).

### Study setting

The Health and Demographic Surveillance System (HDSS) has been running in Matlab since 1966. Matlab is a rural area, located 55 km southeast of Dhaka. icddr, b has been collecting vital statistics (live births, stillbirths, miscarriages, deaths, marriages/dissolution and in and out-migration) through community health research workers (CHRWs) since 1966 [19]. The CHRWs collect vital demographic data by visiting each household on a bi-monthly basis. At each visit, CHRWs complete vital event registration forms.

**Fig. 1:**
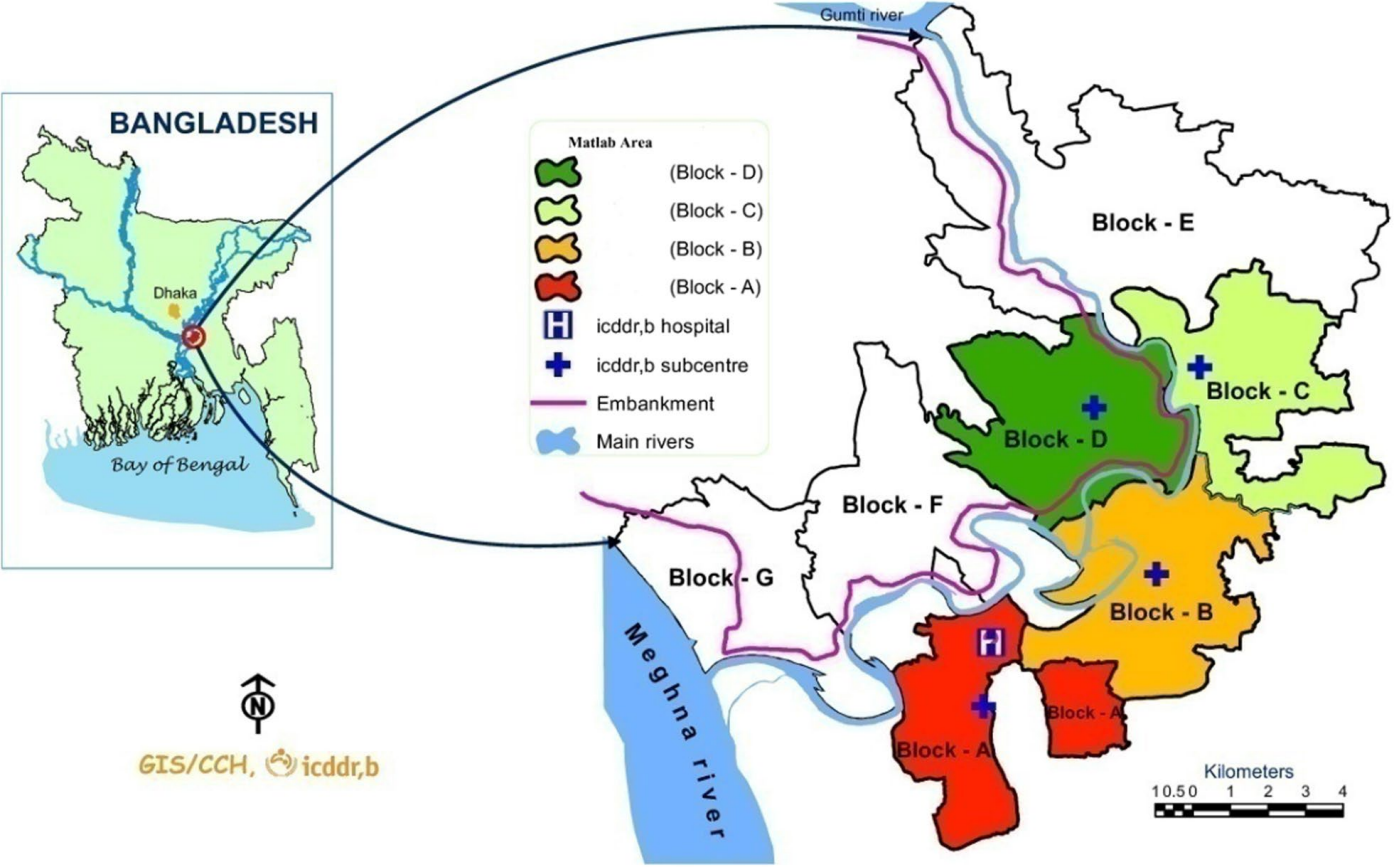
Matlab study setting

The Matlab HDSS area is divided into two parts as shown in Fig. 1: the icddr,b service area (ISA: administrative blocks A, B, C & D) and the government service area (GSA: administrative blocks E, F & G), covering 142 villages since 1987. In 2007, the Maternal, Neonatal and Child Health (MNCH) Project was embedded in the on-going MCH -FP Project in the ISA and has worked to increase the proportion of facility-based deliveries and to introduce an evidence-based maternal and neonatal package which provides services throughout the pregnancy continuum till 6 months after delivery [18]. In addition to documenting vital events, CHRWs in the ISA are trained to provide basic maternal health care, information on contraception and contraceptives, and immunizations for mothers and children under the MCH-FP Project. Each administrative block in the ISA serves a population of about 27,000 and each has subcenter hospital staffed by midwives who provide 24/7 delivery care and related services. These subcenter hospitals are directly linked with the MCH—FP clinics in the Matlab Township, which is staffed by doctors and nurses to provide basic obstetric care round the clock [20]. In the Matlab hospital, every delivery follows standard clinical guidelines prepared by the Obstetrics and Gynaecology Society of Bangladesh (OGSB) & Lamb Hospital [21].

In the GSA administrative block (E,F, and G), there is a population of 115,000, and only standard government services are provided. The GSA has three government Family Welfare Centres (FWCs) where a family welfare visitor (FWV) is posted to provide MNCH services ANC, postnatal care (PNC), delivery care, TT injections and child vaccinations to the respective population. These services are available 24/7. If a pregnancy is complicated and out of a FWV’s capacity to deal with they refer the mother to the Upazilla Health Complex (UHC) which is the nearest higher referral point for each FWC. In both areas, pregnancies are identified by a pregnancy strip test using a morning urine sample.

Data collection in icddr,b service area (ISA) and Government service area(GSA):

The HDSS collects routine data from both the ISA & GSA. There are two groups of CHRWs in the ISA: Surveillance CHRWs (*n* = 43) and Service CHRWs (*n* = 41). In the ISA, both types of CHRWs are available; in the government area, only surveillance CHRWs are available. Service CHRWs collect data through monthly visits to each household. Surveillance CHRWs visit each household every two months. CHRWs collect data using a register book named the “Service Record Book” (SRB) and these records are collected electronically using handheld tablets. In the ISA, CHRWs collect data on reproductive events (menstrual status, pregnancy and outcome status, lactation status, contraceptive use, under-five children’s diarrhea and pneumonia history of last two weeks), the immunization status of eligible women and their under-5 children. All services provided to eligible mothers and children are recorded in a family visit record (FVR) book for every household in the ISA. In each FVR, all data are recorded for each member of the household. Each CHRW carries these electronic Tablets with her during her field visits covering 24 households in a month and 410 couples in 18 months. During the CHRW visit, if a woman is found in her missing period for one and half months, then the CHRW performs a urine test for pregnancy and gives her a Health Service card and requests the woman to visit the subcenter clinic (each block has one subcenter clinic) for further care. At the subcenter, the midwives provide a full range of services: antenatal care and postnatal care, counselling on pregnancy risks, deliveries, keeping of all records, and referral of patients to the Matlab hospital if required. Midwives are fully qualified nurses or midwives and CHRWs have at least passed class ten [19]. icddr,b has deployed 6 CHRWs for each block solely for surveillance data collection since 1966. Each CHRW completes data collection from 1200 households every two months.

### Quality of the data

Each CHRW area is annually assigned at the beginning of the year. Each month all CHRWs sit together, in both the icddr,b and government service areas to update their registrar books. The supervisor routinely provides spot checks of a 2% sample. After going through three tiers of supervision by field research supervisors (FRSs) and field research officers (FRO), and a senior manager, respectively, on the field and then processing through an error detecting program at the central office. All cleaned data are stored within the longitudinal electronic data system and checked with a set of validation before final storage.

### Data analysis

Quantitative data were analyzed using SPSS 23 statistical software. The outcome variables were maternal health-seeking behavior among adolescent mothers. Healthy behavior was defined as 1, Attending 4 + ANC visits and 2, having a facility-based delivery in either a government or non-government facility. The independent variables covered socio-demographic and general characteristics of the mothers as well as the distance to the nearest facility. Economic status was measured in asset quintiles rather than in terms of income or consumption in the study area [22, 23]. Assets included durable goods (e.g., table, chair, watch, television, or bicycle), housing facilities (e.g., type of toilet, or source of drinking water), housing materials (e.g., type of wall or roof), and possession of farming land. Socioeconomic survey data of the year 2014 was used to construct asset quintiles. Socio-demographic differences between these two service areas were measured through chi-square tests for categorical variables and t-tests for the quantitative variables. The distribution of 4 + ANC visits and facility-based deliveries among adolescent women from 2007 to 2015 was explored for both the areas. The predictors associated with healthy pregnancy behaviors and having a facility delivery were determined through binary logistic regression analysis and adjusted for socio-demographic variables. Statistical significance was defined as *p* values of < 0.05.

## Results

The socio-demographic characteristics of adolescent mothers are described in Table 1.

**Table 1:** Socio-demographic characteristics of adolescent mothers in both icddr,b service area (ISA) and Government service area (GSA)

Socio-demographic variables	ISA (2892)	GSA (2104)		*p* value	
	*n* (%)	*n* (%)			
Maternal education					
No education	83 (2.9)		74 (3.5)		< 0.001*
Primary	472 (16.3)		431 (20.5)		
Above primary	2337 (80.8)		1599 (76.0)		
Paternal education					
No education	1270 (43.9)		905 (43.0)		0.008*
Primary	600 (20.7)		511 (24.3)		
Above primary	1022 (35.3)		688 (32.7)		
Religion					
Islam	2570 (88.9)		1963 (93.3)		< 0.001*
Hindu	322 (11.1)		141 (6.7)		
Asset score					
Lowest	454 (15.7)		324 (15.4)		0.090
Second	545 (18.8)		403 (19.2)		
Middle	525 (18.2)		417 (19.8)		
Fourth	644 (22.3)		499 (23.7)		
Richest	724 (25.0)		461 (21.9)		
Parity					
Nullipara	46 (1.6)		43 (2.0)		< 0.001*
1	2749 (95.1)		1922 (91.3)		
2	97 (3.4)		139 (6.6)		
Place of delivery					
Home	549 (19.0)		1262 (60.0)		< 0.001*
Facility	2343 (81.0)		842 (40.0)		

*Indicates that the results are significant at *p* value < 0.05

Among the 4,996 adolescent mothers, more than 90% had completed at least primary education or higher, which is greater than the percentage of father's primary and higher education level. In both areas, adolescent mothers are predominantly Muslim and most of the adolescent mothers had a parity of one. Facility deliveries were more than double in the icddr,b service area (ISA) relative to the government service area (GSA).

**Fig. 2:**
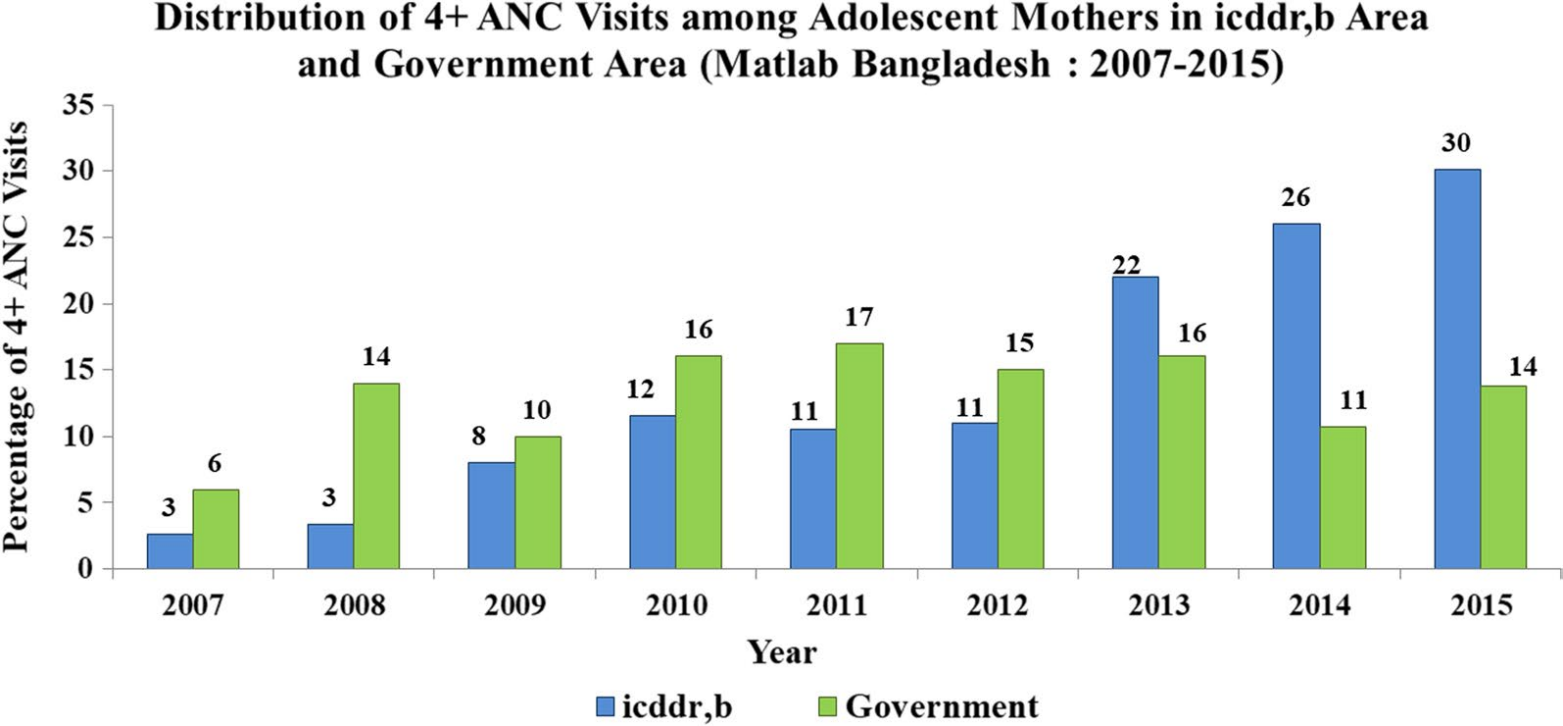
Distribution of 4 + ANC visits in both icddr,b service area and Government service area among adolescent mothers (Matlab Bangladesh: 2007–2015)

Figure 2 shows the distribution of 4 + ANC visits in both ISA and GSA. The rate was higher in GSA till 2012 but started to fall after that the year the 4 + ANC attendance became higher in ISA (22%) than the GSA (16%) on 2013 the gap in service coverage has continued to increase since 2014 onward.

Table 2 shows the results from bivariate and multivariate findings on the determinants of 4 + ANC among adolescent mothers. The adjusted model included area of residence (ISA vs. GSA), maternal education, paternal education, religion, asset score, repeated pregnancy, and distance from nearest facility.

**Table 2:** Factors associated with 4 + ANC visits: results from bivariate and multivariate analysis

	4 + ANC visits			Adjusted effects		
	No (*N* = 4292)	Yes (*N* = 704)	*p* value	Adjusted OR	95% CI	*p* value
	*n* (%)	*n* (%)				
Service area						
icddr,b service area (ISA)	2467 (85.3)	425 (14.7)	0.150	Ref	–	–
Government service area (GSA)	1825 (86.7)	279 (13.3)		0.57	0.49–0.66	< 0.001*
Maternal education						
No education	146 (93.0)	11 (7.0)	< 0.001*	0.39	0.20–0.72	0.003*
Primary	807 (89.4)	96 (10.6)		0.72	0.56–0.91	0.007*
Above primary	3339 (84.8)	597 (15.2)		Ref	–	–
Paternal education						
No education	1852 (85.1)	323 (14.9)	0.006*	0.49	0.43–0.57	< 0.001*
Primary	987 (88.8)	124 (11.2)		0.48	0.38–0.59	< 0.001*
Above primary	1453 (85.0)	257 (15.0)		Ref	–	–
Religion						
Islam	3918 (86.4)	615 (13.6)	0.001*	Ref	–	–
Hindu	374 (80.8)	89 (19.2)		0.95	0.73–1.22	0.673
Asset score						
Lowest	693 (89.1)	85 (10.9)	< 0.001*	0.31	0.24–0.40	< 0.001*
Second	831 (87.7)	117 (12.3)		0.31	0.25–0.39	< 0.001*
Middle	814 (86.4)	128 (13.6)		0.33	0.27–0.41	< 0.001*
Fourth	977 (85.5)	166 (14.5)		0.34	0.28–0.40	< 0.001*
Richest	977 (82.4)	208 (17.6)		Ref	–	–
Repeated pregnancy						
Yes	272 (85.8)	45 (14.2)	0.956	0.71	0.51–0.99	0.049*
No	4020 (85.9)	659 (14.1)		Ref	–	–

*Indicates that the results are significant at *p* value < 0.05

Bivariate findings revealed that maternal education, paternal education, religion, and asset scores were significantly related to 4 + ANC visits. In total 704 adolescent mothers from both ISA and GSA had received 4 + ANC. It is seen that the percentage of mothers from ISA (14.7%) who received 4 + ANC was (*p* value < 0.05) higher than the mothers from GSA (13.3%). Only 15.2% of adolescent mothers with the above primary education received 4 + ANC from both areas.

Table 2 also shows that the adjusted odds of 4 + ANC visits among adolescent mothers were 43% lower in GSA (OR = 0.57, 95% CI 0.49–0.66, *p* value < 0.05) compared to that of ISA. Adolescent mothers with no education (OR = 0.39, 95% CI 0.20–0.72, *p* value < 0.05) and primary education (OR = 0.72, 95% CI 0.56–0.91, *p* value < 0.05) were less likely to receive 4 + ANC compared to adolescent mothers having above primary education. Similar trends were found for paternal education. People from Hindu communities (OR = 0.95, 95% CI 0.73–1.22) were less likely to receive four or more ANC than Muslims though the results were not significant. Asset scores were also found to be a significant determinant for receiving 4 + ANC. Poorest adolescent mothers were less likely to receive 4 + ANC (OR = 0.31, 95% CI 0.24–0.40, *p* value < 0.05) compared to richest adolescent mothers. Similar behavior found in adolescent women of other asset score groups compared to the richest group.

**Fig. 3:**
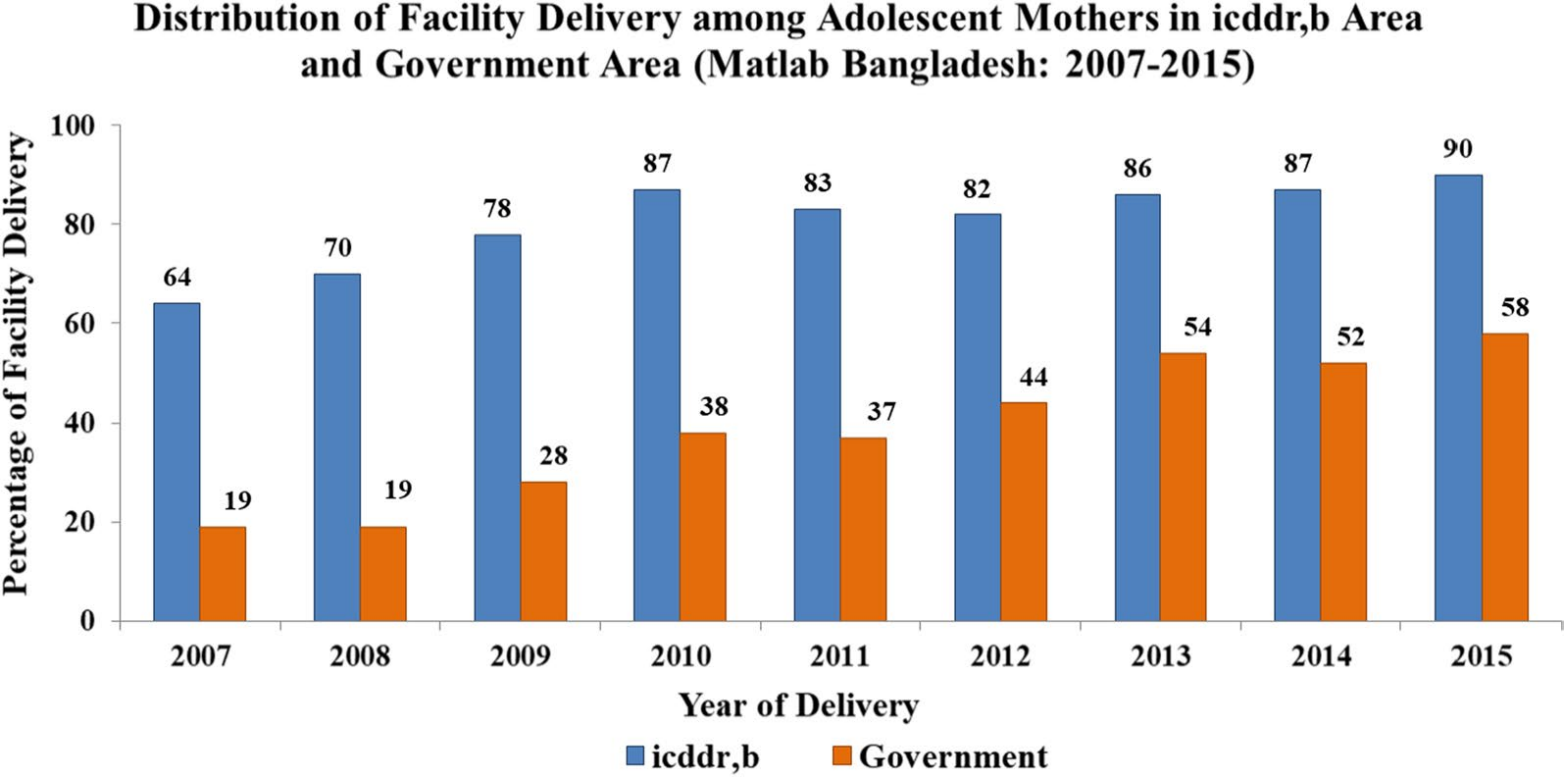
Distribution of facility delivery among adolescent mothers in icddr,b service area and Government service area (Matlab Bangladesh: 2007–2015)

Figure 3 shows the distribution of facility deliveries among adolescent mothers in both ISA and GSA. The percentage of adolescent mothers having facility deliveries in the ISA was consistently higher than in the GSA. Facility deliveries among adolescent mothers increased in both ISA and GSA between 2007 and 2015.

Table 3 illustrates the determinants associated with receiving facility delivery in ISA and GSA.

**Table 3:** Factors associated with facility delivery: results from bivariate and multivariate analysis

	Facility delivery			Adjusted effects		
	No (*N* = 1811)	Yes (*N* = 3185)	*p* value	Adjusted OR	95% CI	*p* value
	*n* (%)	*n* (%)				
Service area						
icddr,b service area (ISA)	549 (19.0)	2343 (81.0)	< 0.001*	6.63	5.85- 7.52	< 0.001*
Government service area (GSA)	1262 (60.0)	842 (40.0)		Ref	–	–
Maternal education						
No education	67 (42.7)	90 (57.3)	< 0.001*	0.77	0.53–1.11	0.165
Primary	474 (52.5)	429 (47.5)		0.51	0.43–0.61	< 0.001*
Above primary	1270 (32.3)	2666 (67.7)		Ref	–	–
Paternal education						
No education	735 (33.8)	1440 (66.2)	< 0.001*	1.05	0.92–1.20	0.464
Primary	519 (46.7)	592 (53.3)		0.73	0.62–0.87	0.001*
Above primary	557 (32.6)	1153 (67.4)		Ref	–	–
Religion						
Islam	1669 (36.8)	2864 (63.2)	0.009*	Ref	–	–
Hindu	142 (30.7)	321 (69.3)		1.13	0.89–1.44	0.307
Asset score						
Lowest	348 (44.7)	430 (55.3)	< 0.001*	0.55	0.45–0.67	< 0.001*
Second	362 (38.2)	586 (61.8)		0.71	0.59–0.86	< 0.001*
Middle	388 (41.2)	554 (58.8)		0.59	0.49–0.70	< 0.001*
Fourth	399 (34.9)	744 (65.1)		0.75	0.64–0.88	< 0.001*
Richest	314 (26.5)	871 (73.5)		Ref	–	–
No. of ANC visits						
Less than 4	1647 (38.4)	2645 (61.6)	< 0.001*	Ref	–	–
4 +	164 (23.3)	540 (76.7)		2.04	1.67–2.49	< 0.001*
Repeated pregnancy						
Multiple	128 (40.4)	189 (59.6)	0.114	0.90	0.69–1.18	0.450
Single	1683 (36.0)	2996 (64.0)		Ref	–	–

*Indicates that the results are significant at *p* value < 0.05

Bivariate findings demonstrated that 3185 adolescent mothers from both ISA and GSA accessed facility deliveries. Service area, maternal education, paternal education, religion, asset score, and increased number of ANC visits were found to be significant predictors of facility-based deliveries among adolescent mothers (*p* value < 0.05). 80.0% of adolescent mothers in ISA had accessed facility delivery whereas in GSA only 40.0% had accessed facility deliveries. Less than 50% of primary educated adolescent mothers’ and approximately 67.7% of adolescent mothers’ with higher education receive facility delivery. In addition, 53.3% of fathers with primary education and 67.4% of fathers with higher education assisted their wives to receive facility based deliveries. 76.7% of adolescent mothers from both areas who had received 4 + ANC also received facility-based delivery care.

The adjusted odds of receiving facility-based delivery among adolescent mothers was almost 6 times higher in ISA compared to that of GSA (OR = 6.63, 95% CI 5.85–7.52), *p* value < 0.05). Poorest adolescent mothers (OR = 0.55, 95% CI 0.45–0.67, *p* value < 0.05) were less likely to receive facility deliveries compared to the richest. Other asset score groups of adolescent mothers also have shown the same behavior compared to the richest group in receiving facility delivery. Adolescent mothers who received 4 + ANC during pregnancy were more likely to receive facility delivery service compared to those who did not receive 4 + ANC (OR = 2.04, 95% CI 1.67–2.49, *p* value < 0.05) (Table 3).

To visualize the effect of the practice of 4 + ANC visits on receiving facility delivery in ISA and GSA separately we have done two logistic regression analyses using data from ISA and GSA separately. Findings showed (data not shown) that adolescent mothers who received 4 + ANC during pregnancy were more likely to receive facility delivery services compared to those who did not received 4 + ANC in both ISA (OR = 3.33, 95% CI 2.39–4.62, *p* value < 0.05) and GSA (OR = 1.96, 95% CI 1.52–2.53, *p* value < 0.05).

## Discussion

This study documented that the uptake of 4 + ANC visits and facility-based deliveries are higher among adolescent mothers residing in the icddr,b area relative to the government area. The inbuilt nature of the MNCH service delivery in the icddr,b area could be a factor contributing to this [18]. We examined the data of very young adolescents (10–14 year) and found the distribution of VYA sample in this group to be only 0.4%, so we did not perform the analysis by very young adolescent and older adolescent separately (15–19 year). Receiving 4 + ANC visits during pregnancy is an important predictor of adolescent mothers delivering their babies in facilities for both areas; however, the association between 4 + ANC visits and receiving facility delivery were stronger in ISA than GSA in this study.

Four or more ANC visits were found to be more likely in ISA than GSA. The ANC rate is much higher than other reported studies [19, 24]. This is probably attributable to the quality of ANC services, which have improved patient knowledge and recognition of pregnancy danger signs, and referral. These factors support increasing 4 + ANC visits and facility delivery in the ISA compare to GSA, as was observed in a 2011 Matlab MNCH study [18]. For this study, adolescent mothers who practice 4 + ANC uptake during pregnancy are more likely to receive facility delivery service which is similar to other developing countries [25]. In ISA, the approach to providing care for mothers, newborns, and children is more integrated than in GSA. In the former, maternal health services are often well received, provided free of charge, and without any hidden costs, which may not be the case in GSA The details of services are mentioned in elsewhere [18, 26] ADDIN EN.CITE [18, 26]. This might be a probable cause of more quality services are available in ISA than GSA.

The community skilled birth attendant (CSBA) initiative, which began in Bangladesh in 2003, may be to blame for GSA's poor performance in comparison with ISA. The CSBA programme trained the Female Health Assistants (FHA) from DGHS and Family Welfare Assistant (FWA) from DGFP for 6 months on safe delivery. They used to attend delivery at home which causes detract their day to day home visits for organizing MNCH services [27]. This was also reported increasing the number of CSBA and also decreasing the household visit by FWA and FHA in BDHS 2016 [24]. But this was not case in ISA. So, lack of contact and communication of the GSA filed workers rather busy with home delivery might reduce the performances for ANC and delivery care in GSA.

Significant determinants of facility delivery in both ISA and GSA were maternal education, paternal education, higher asset scores, religion, number of ANC visits, and distance from nearest facility. However, the percentage receiving facility-based delivery was higher among ISA compared to GSA even when controlling for these factors. This suggests that icddr,b interventions in the ISA have contributed to improved adolescent maternal health behavior.

As per earlier studies, educated mothers are more likely to take advantage of public health care services, seek high-quality services and have greater ability to use health care inputs that offer improved care than women with no education [28, 29]. Findings revealed an important impact of maternal education on the practice of healthy behaviors among adolescent mothers for this study. However, this study suggests that adolescent mothers, whose husbands had higher educational levels, were more likely to receive maternal health services than others were. These findings are similar to other studies [30, 31].

For this study, the Hindu community was less likely to obtain 4 + ANC visits and but more likely to receive facility delivery than Muslim community, though the result was insignificant (which might be a result of sampling fluctuation). These findings are inconsistent with that of an earlier study, which highlighted that Hindu and Muslim women are similar in availing of delivery care [32]. The findings revealed inequities in receiving 4 + ANC and facility delivery by socioeconomic strata in Matlab Bangladesh. The economic barriers to maternal health care are still a key determinant to accessing the services in the study area. Richest people were more likely to receive 4 + ANC visits as well as facility delivery than poor in both areas which are a common scenario across different countries of the developing world [33, 34]. This finding suggests financial barriers may influence health service utilization for adolescent mothers to achieve universal health coverage in the context of Bangladesh [35].

### Strengths and limitations

Data from Matlab HDSS has been criticized for not being representative of other rural areas of Bangladesh because of its many and long-term interventions in the field of health, population, and nutrition [36]. Additionally, the current Matlab data collection system does not allow monitoring of the WHO recommended 8 ANC visits. Finally, it has been noted that GSA CHRWs have a much larger catchment population than ISA CHRW’s, which may result in less robust GSA data. This type of longitudinal data on adolescent health is not available in other part of Bangladesh, which accounts for the uniqueness in the results analysis of Matlab HDSS surveillance data. The rigor of the data quality procedures, long-standing follow-up in nature of the HDSS has provided a unique opportunity to produce authentic results from the analysis [19].

## Conclusion

Enhanced 4 + ANC visits and a higher prevalence of facility deliveries indicate that interventions in the ISA are supporting adolescent mothers’ access to maternal care. Interventions implemented in ISA, if scaled, have the potential to ensure that every adolescent mother received the best standard of care, regardless of economic status and residence of pregnant women. Reducing the prevalence of adolescent pregnancies, and ensuring all pregnant adolescents reach care will support for Bangladesh’s national strategic guidelines, and the achievement of SDG 3.8 which refers essential health service should be available to all respective persons by 2030 [35].

## Data Availability

Data contain potentially identifying or sensitive information from delivering women. However, “Data can be available on request”. The data request should be submitted to the Research Administration (RA) of iccdr,b and will be assessed by the corresponding Ethics committee named institutional Review Board of icddr,b. As a supplementary information, we have added approved protocol providing the study title and protocol number (PR-17087) against which data access application should be made. Please visit https://www.icddrb.org/ dmdocuments/icddrb%20Data%20Access% 20Policy.pdf for additional information. Data requests are evaluated by icddr,b’s Data Repository Committee (DRC) and the Research Administration (RA) serves as the Secretariat of the DRC. The key contact person of RA at present is Ms. Armana Ahmed, Lead (A), RA at aahmed@icddrb.org If the data request is considered justifiable by the DRC then RA will share the anonymous data with the applicant. Moreover, for any particular clarification of the research findings that is documented in this article, queries can be directed to the primary author of this article or to the corresponding author. Both of them can be accessed at draminur@icddrb.org. The email correspondence regarding data access could be done at the executive director office at dircetor@icddrb.org.

## Abbreviations

ANC: Antenatal care
BDHS: Bangladesh Demographic And Health Survey
CHRW: Community Health Research Worker
CI: Confidence interval
CSBA: Community skilled birth attendant
DGFP: Director General of Family Planning
DGHS: Director General of Health Service
FHA: Female Health Assistant
FRO: Field Research Officer
FRS: Field Research Supervisor
FVR: Family Visit Record
FWA: Family Welfare Assistant
FWV: Family Welfare Visitor
GIS: Geographical Information System
GSA: Government Service Area
HDSS: Health and Demographic Surveillance System
icddr,b: International Centre for Diarrhoeal Research, Bangladesh
ISA: Icddr,b service area
LMIC: Lower-to-middle-income country
MCH-FP: Maternal and Child Health and Family Planning
MMR: Maternal mortality ratio
MNCH: Maternal Neonatal and Child Health
OGSB: Obstetrics and Gynecology Society of Bangladesh
OR: Odds ratio
SDG: Sustainable Development Goal
SPSS: Statistical Package for the Social Sciences
SRB: Service Record Book
TFR: Total fertility rate
UHC: Upazila Health Complex
UHFWC: Upazila Health and Family Welfare Centre
WHO: World Health Organization

## References

[1] Dangal G (2005). An update on teenage pregnancy. Internet J Gynecol Obstetr.

[2] Raatikainen K (2005). Good outcome of teenage pregnancies in high-quality maternity care. Eur J Public Health.

[3] Wiliamson N. Motherhood in childhood: facing the challenge of adolescent pregnancy. State of world population 2013;2013.

[4] Abu-Heija A, Ali AM, Al-Dakheil S (2002). Obstetrics and perinatal outcome of adolescent nulliparous pregnant women. Gynecol Obstet Invest.

[5] Organization WH (2016). WHO recommendations on antenatal care for a positive pregnancy experience.

[6] Rahman M, Islam R, Islam AZ (2008). Rural-urban differentials of utilization of ante-natal health-care services in Bangladesh. Health Policy Dev.

[7] Islam MM, Masud MS (2018). Determinants of frequency and contents of antenatal care visits in Bangladesh: assessing the extent of compliance with the WHO recommendations. PLoS ONE.

[8] Huq NL (2015). Effect of an integrated maternal health intervention on skilled provider’s care for maternal health in remote rural areas of Bangladesh: a pre and post study. BMC Pregnancy Childbirth.

[9] Finlayson K, Downe S (2013). Why do women not use antenatal services in low-and middle-income countries? A meta-synthesis of qualitative studies. PLoS Med.

[10] Jacobs B (2011). Addressing access barriers to health services: an analytical framework for selecting appropriate interventions in low-income Asian countries. Health Policy Plan.

[11] Deo KK (2015). Barriers to utilization of antenatal care services in Eastern Nepal. Front Public Health.

[12] El Arifeen S (2014). Maternal mortality in Bangladesh: a Countdown to 2015 country case study. The Lancet.

[13] Jolly MC (2000). Obstetric risks of pregnancy in women less than 18 years old. Obstet Gynecol.

[14] Rahman MM (2016). Maternal pregnancy intention and professional antenatal care utilization in Bangladesh: a nationwide population-based survey. PLoS ONE.

[15] Siddique AB (2018). Antenatal care in rural Bangladesh: gaps in adequate coverage and content. PLoS ONE.

[16] MNCH D. National Strategy for Adolescent Health 2017–2030; MCH Services Unit, Directorate General of Family Planning Ministry of Health and Family Welfare, 2016.

[17] Statistical Yearbook for Asia and the Pacific 2017: SDG datasheet.

[18] Rahman A (2011). Effectiveness of an integrated approach to reduce perinatal mortality: recent experiences from Matlab, Bangladesh. BMC Public Health.

[19] Alam N (2017). Health and demographic surveillance system (HDSS) in Matlab, Bangladesh. Int J Epidemiol.

[20] Rahman M et al. Health and demographic surveillance system-Matlab: volume forty seven; registration of health and demographic events 2013;2015.

[21] Begum T (2017). Indications and determinants of caesarean section delivery: evidence from a population-based study in Matlab, Bangladesh. PLoS ONE.

[22] Gwatkin DR (2007). Socio-economic differences in health, nutrition, and population within developing countries.

[23] Filmer D, Pritchett LH (2001). Estimating wealth effects without expenditure data—or tears: an application to educational enrollments in states of India. Demography.

[24] Research NIoP et al. Bangladesh demographic and health survey, 2014. 2016: NIPORT.

[25] Kawungezi PC (2015). Attendance and utilization of antenatal care (ANC) services: multi-center study in upcountry areas of Uganda. Open J Prev Med.

[26] Mridha MK, Anwar I, Koblinsky M (2009). Public-sector maternal health programmes and services for rural Bangladesh. J Health Popul Nutr.

[27] Ahmed T, Jakaria SM (2009). Community-based skilled birth attendants in Bangladesh: attending deliveries at home. Reprod Health Matters.

[28] Shahjahan M (2017). Antenatal and postnatal care practices among mothers in rural Bangladesh: a community based cross-sectional study. Midwifery.

[29] Singh L, Rai RK, Singh PK (2012). Assessing the utilization of maternal and child health care among married adolescent women: evidence from India. J Biosoc Sci.

[30] Singh PK (2012). Determinants of maternity care services utilization among married adolescents in rural India. PLoS ONE.

[31] Banke-Thomas OE, Banke-Thomas AO, Ameh CA (2017). Factors influencing utilisation of maternal health services by adolescent mothers in Low-and middle-income countries: a systematic review. BMC Pregnancy Childbirth.

[32] Rahman M (2011). Noninstitutional births and newborn care practices among adolescent mothers in Bangladesh. J Obstet Gynecol Neonatal Nurs.

[33] Geta MB, Yallew WW. Early initiation of antenatal care and factors associated with early antenatal care initiation at health facilities in Southern Ethiopia. Adv Public Health. 2017;2017.

[34] Fagbamigbe AF, Idemudia ES (2015). Barriers to antenatal care use in Nigeria: evidences from non-users and implications for maternal health programming. BMC Pregnancy Childbirth.

[35] Organization WH (2016). World health statistics 2016: monitoring health for the SDGs sustainable development goals.

[36] Rasheed F, Karim E (2000). STD research and policy formulation. Lancet.

